# Stem Cells in the Path of Light, from Corneal to Retinal Reconstruction

**DOI:** 10.3390/biomedicines9080873

**Published:** 2021-07-23

**Authors:** Ovidiu Samoila, Lacramioara Samoila

**Affiliations:** 1 Ophthalmology Department, University of Medicine and Pharmacy “Iuliu Hatieganu”, 400006 Cluj-Napoca, Romania; 2 Clinica Vedis, 400371 Cluj-Napoca, Romania; lacri_totu@yahoo.com

**Keywords:** stem cells, transplantation, cell cultivation, cornea, retina, tissue engineering

## Abstract

The future of eye reconstruction invariably includes stem cells transplantation. Corneal limbus, corneal stroma, trabeculum, retinal cells, optic nerve, and all structures that are irreversibly damaged and have no means to be repaired or replaced, through conventional treatment or surgery, represent targets for stem cell reconstruction. This review tries to answer the question if there is any clinical validation for stem therapies, so far, starting from the cornea and, on the path of light, arriving to the retina. The investigation covers the last 10 years of publications. From 2385 published sources, we found 56 clinical studies matching inclusion criteria, 39 involving cornea, and 17 involving retina. So far, corneal epithelial reconstruction seems well validated clinically. Enough clinical data are collected to allow some form of standardization for the stem cell transplant procedures. Cultivated limbal epithelial stem cells (CLET), simple limbal epithelial transplant (SLET), and oral mucosa transplantation are implemented worldwide. In comparison, far less patients are investigated in retinal stem reconstructions, with lower anatomical and clinical success, so far. Intravitreal, subretinal, and suprachoroidal approach for retinal stem therapies face specific challenges.

## 1. Introduction

The vision system is the most important and complex sense for the perception of the world. Most of the information we receive, comes in some form of light. Mechanical, optical, neurological, chemical, and electrical properties of the eye need to be kept in balance, for us to receive a clear image of the surroundings. The path of light goes through transparent lenses until it reaches the retina. Regeneration medicine aims to re-establish the transparency of Pthese lenses, cornea especially, and the normal architecture of retina.

The presence of stem cells on the ocular surface was accepted decades ago, starting with the research conducted by Davanger and Evensen, in 1971 [1], who proposed that limbal epithelial stem cells (LESC) reside in the palisades of Vogt at the limbus, at the periphery of the cornea. Dua, in 2005 [2], reported the presence of limbal epithelial crypts that harbour LESC. Conjunctival epithelium may also harbour stem cells, but the precise location is still under debate. Slow cycling cells were found in the fornix, but also in the palpebral conjunctiva. Boulton [3] suggests that each region of the conjunctiva may have its own niche. Since then, stem cells have been searched in other regions of the eye. It is not clear if neural retina or retinal pigment epithelium have any regenerative capabilities. Muller glial cells in the retina may have stem properties. Hematopoietic stem cells may play a role in neovascularization, as an endothelial precursor, in retinal diseases such as diabetic retinopathy [3,4].

The potential to reconstruct any damaged structure made stem cells an innovative therapy to treat various eye diseases, either on the surface of the eye or at the back of the eye, at the retina. Regardless of the natural presence of adult stem cells at the site needing treatment, various cultivated stem cells or cell suspensions were applied, injected or glued, in different locations of the eye, in the hope that they proliferate to replace the damaged tissue, borrowing the properties of the surroundings and of the cells intended to be replaced.

Stem cells are regarded as a promise for any type of tissue restoration, being able to proliferate, migrate, and differentiate into various progenitor cells. Cultivated limbal epithelial stem cells (CLET), simple limbal epithelial transplant (SLET), with allogeneic variations, are currently used for ocular surface reconstructions. Oral mucosa is another source of stem cells (replacing the corneal epithelium with cultivated oral mucosal epithelial sheet transplantation—COMET or cultivated autologous oral mucosa epithelial cell-sheet—CAOMECS). The retinal approach uses rather suspensions of stem cells, deployed either internally (intravitreal or subretinal—via pars plana vitrectomy) or externally (placing the stem cells into the suprachoroidal space, via deep sclerectomy). Lately, stem cells supported by a matrix were also investigated.

Stem cell therapy uses three types of cells: Pluripotent stem cells, fetal stem cells, and adult stem cells. Adult type and pluripotent are the most used in the eye. Other than cornea, sources for stem cells in eye therapies are the human teeth (dental pulp), hair follicle, bone marrow, adipose tissue or umbilical cord [5]. These are sources for human embryonic stem cells (hESCs), mesenchymal stem cells (MSCs), which include bone marrow MSCs (BMSCs) or adipose-derived MSCs (ADSCs). When compared to BMSCs, ADSCs have the advantages of easier harvest from donors, faster expansion, more protein secretion, and higher immunomodulatory capacity [6]. The stromal vascular fraction (SVF) derived from adipose tissue contains heterogeneous cell populations such as mesenchymal stem cells, preadipocytes, endothelial cells, pericytes, T cells, and M2 macrophages. SVF-derived mesenchymal stem cells can be easily expanded in vitro and have the potential to create diverse lineages of cells [7]. Adult somatic cells of various origins (fibroblasts, blood cells) can be genetically engineered to produce induced pluripotent stem cells (iPSCs), which show similar proliferative and pluripotency properties to hESCs [8].

## 2. Materials and Methods

The subject of stem cells is fascinating, and stem cells therapies hold great promise for the restoration of vision in people without any other hope. However, there is a difference in approach regarding stem cells on the ocular surface and on the retina. On the ocular surface there are stem cells already residing there, and the placement of any other stem cells seems somehow natural. On the other hand, the retinal reconstruction (especially the neural retina) implies the placement of proliferative cells in a place where there are no active stem cells anymore, after birth. We wanted to answer the question if there is any clinical validation of stem therapies, so far, starting from the cornea, and, on the path of light, arriving to the retina.

The authors underwent an extensive literature search, to identify any significant clinical application of stem cell therapies in the eye.

The search criteria on PubMed (with search terms: “eye; stem cell; transplant”) revealed 2385 results in the last 10 years. Applying restrictions: Humans, clinical studies, number of eyes included (at least 5 treated eyes), resulted in 56 clinical studies between 2010–2020, 39 for corneal reconstruction and 17 for retina. Clinicaltrials.gov site was also verified for completed clinical trials with published results, with the same criteria.

## 3. Results

Between 2010 and 2020, we found 39 clinical studies regarding corneal reconstruction, matching criteria. The summary of the studies, including author, year, type of clinical study, concept (regarding cultivation of stem cells and surgery), and outcome (anatomical and functional) are presented in Table 1.

In total, about 1479 eyes received stem cells on the ocular surface. The ethiology of LSCD was diverse: Burns (either chemical or thermal), surgical, infectious (keratitis), contact lens abuse, immunological (Stevens Johnson, pemphigoid) or congenital (aniridia). Some studies focused on replacing cells inside the stroma, in keratoconus [9,10]. CLET was used in 12 studies (autologous cells from the same eye or from contralateral eye), allogeneic CLET (AlloCLET) in 13 studies (allogeneic stem cell, cadaveric source), SLET in seven studies, allogeneic SLET in one study, ADSC injected in stroma in two studies and cultivated allogeneic MSC (MSCT) in one study. Oral mucosa was transplanted in seven studies, either in the form of COMET (five studies) or CAOMECS (two studies). Overall anatomical success varied from 29% to 100%. Most of the studies reported 60–80% anatomical success rate. The anatomical outcome was generally defined as a success if a stable corneal epithelium, transparent and avascular, was generated. Opaque, vascularized epithelium, with persistent epithelial defects, was noted as failure. The functional outcome was represented as visual acuity (either LogMAR or decimal system). Results of in vivo confocal microscopy (IVCM), impression cytology (IC), corneal OCT or DNA analysis, where available, were presented.

Retinal reconstruction was described in 17 clinical studies between 2010 and 2020, matching inclusion criteria. Table 2 presents the summary for 11 of these studies, including author, year, number of patients and eyes treated, surgery approach (external or internal, intravitreal or subretinal), type of stem cells used, and outcome, both anatomical/clinical and functional. The remaining of the retinal studies are presented in Table 3. All the six articles are part of the stem cell ophthalmology treatment study (SCOTS), with partial results published by the same team (Weiss et al.). In SCOTS, the study design is similar for different pathologies (dominant optic atrophy, retinitis pigmentosa, Usher syndrome, AMD, Leber neuropathy, nonarteritic optic neuropathy) and implied a combination of injected BMSC, retrobulbar (RB), subtenon (ST), intravenous (IV), intravitreal (IVIT), and intraocular via pars plana vitrectomy (Arms 1, 2, and 3). Number of patients, follow up, de facto stem delivery (the choice between Arms 1, 2, and 3) and clinical outcomes are presented.

In total, 211 eyes were treated with stem cells for various retinal and optic nerve disease: Age related macular degeneration (AMD), Stargardt dystrophy, retinitis pigmentosa, vascular disease, optic nerve atrophy or Leber neuropathy. Different types of stem cells (adipose, mesenchymal or iPSC) were either injected intravitreally, placed in subretinal space (with pars plana vitrectomy) or suprachoroidal space (with external approach of the eye). The anatomical and clinical outcome was evaluated by retinal OCT or multifocal ERG (mERG). The presence of the implanted material on OCT was considered a success, together with improvements in mERG, visual field (investigated with microperimetry or automated perimetry), and visual acuity. For better interpretation of outcomes, visual field is presented in Table 2 and Table 3 under clinical outcome, while visual acuity is presented separately. Visual outcome varied a lot, from decrease in vision to improvement, depending also on the surgery employed.

## 4. Discussion

Limbal stem cells are the most studied stem cells of the eye. An injury to the ocular surface will activate the stem cells, who proliferate, migrate, and mature into epithelial cells to regenerate the corneal surface. Unfortunately, various conditions destroy LESCs, leading to limbal stem cells deficiency (LSCD), that can be unilateral or bilateral. These conditions include mostly corneal burns, either chemical or thermic, but also Stevens-Johnson syndrome, ocular pemphigoid, ocular surface surgeries, radiation, corneal infections or improper use of contact lenses. Primary causes for LSCD include aniridia, congenital epidermal dysplasia or xeroderma pigmentosum. LSCD is a clinical manifest with loss of vision, while the cornea becomes opaque and vascularized. Epithelial breakdown leads to persistent epithelial defects. Without the limbus barrier, the conjunctival type of epithelium advances into the cornea, changing the normal corneal cells. In vivo confocal microscopy (IVCM) and impression cytology (IC) show goblet cells, characteristic to conjunctiva. Conjunctival immunohistochemical markers appear in the area of conjunctivalization—cytokeratin 13 and 19, Muc5ac and mucin 1, while the corneal phenotype shows cytokeratins 3 and 12 [11]. Factor p63 also identifies LESCs [1].

The management of LSCD improved dramatically over the years: Kenyon and Tseng in 1989 [12] started conjunctival-limbal autograft transplantation (CLAU), then Tsai and Tseng performed kerato-limbal allografting (KLAL). First cultivation techniques from autologous stem cells were introduced in 1997 by Pellegrini et al. [13], and variations on cultivated epithelial limbal transplantation (CLET), have followed [14,15]. A small biopsy from the healthy eye limbus or from an unaffected area of the same eye (1–2 mm in size) is used for in vitro cultivation. The explant or cell suspension (after the trypsinization of the biopsy) are currently used for the culture. Denuded human amniotic membrane (AM) is used as a substrate and different cultivation mediums are added. Amniotic membrane is sometimes replaced with fibrine or a contact lens. Cocultivation with murine 3T3 feeder layers may promote cell proliferation but carry a risk of infection (prion disease). The elimination of feeder layers and replacing fetal bovine serum with human autologous serum, results in a completely xeno-free cultivation technique.

### 4.1. CLET and SLET

Sangwan [16] reported in 2011, the clinical outcome for CLET, performed starting the year 2001, with a minimum follow up for 12 months (on average 36 months). In addition, 71% (142 out of 200 eyes) showed stable, epithelized, avascular corneal surface, while a BCVA improvement of at least two lines, was seen in 60.5% of the patients. CLET reintervention performed by Basu [17] on 50 patients having failed the primary CLET, were proved successful in 66% of the cases, with BCVA showing at least two-lines increase in 76% of the cases.

Feeling that CLET is somehow restricted to some advanced centers worldwide, Sangwan et al. in 2012 [18] developed a technique that placed LESC directly on the injured eye. The novel technique to transplant cells onto the corneal surface, requires no need for complex and time-consuming laboratory cultivation. Simple limbal epithelial transplantation (SLET) involves the harvest of a small amount of limbal tissue from the contralateral eye and the direct transplantation onto the affected eye, after cutting it in smaller pieces. A 2 by 2 mm biopsy is used, divided in 8–10 smaller pieces that are fixed onto AM in the transplanted eye.

The same concept was applied by Vazirani [19] in 2016, for corneal burns (with 83.3% anatomical success rate). In 2017, Kaliki [20] treated seven cases of ocular surface squamous neoplasia undergoing wide excisional biopsy (mean tumor base, 8 mm). The addition of primary SLET resulted in the absence of LSCD at follow-up, in all cases.

SLET was later successfully replicated with the use of allogeneic cells, harvested from cadaveric limbus, in the work of Iyeer et al. in 2017 [21]. Amniotic membrane was used as a substrate for the stem cells, being sutured over the defect. Iyeer et al. fixed the cells in place with fibrin glue and applied a soft contact lens over the transplant. The authors emphasized that the survival of allocells depends on the immunosuppression and should be performed during the first days after the burn, in the wait for a subsequent limbal autograft, after the acute phase of the inflammation diminishes.

### 4.2. Autologous versus Allogeneic

Cadaveric keratolimbal grafts carry an increased risk of immunological rejection. It was hypothesized that cultivated LESCs (allogeneic CLET or AlloCLET) could carry a lower risk. However, immunological rejection and outcomes seemed to be similar in both techniques, as described by various authors (Satake et al. [22] and Parihar et al. [23]), while keratolimbal allograft did not require as many resources as the cultivation technique. Clinical outcomes of AlloCLET were reviewed in depth in 2020 [24].

Qi [25] noted that immune rejection after allogeneic transplantation occurs mainly 6 months after the transplant and should be readily addressed. In addition, 23.8% of the transplanted eyes experienced immune rejection, with corneal opacification, epithelial reaction, and congestion. Moreover, 76.2% maintained an avascular, stable cornea.

Ramirez et al. [26] noted similar anatomical results for autologous and allogeneic CLET. In a small series, 11 AutoCLET and 9 AlloCLET showed similar results in 1 year. The overall anatomical success was 80% in the first 2 years and 75% after 3 years. By IVCM, 80% of eyes improved the epithelial status.

In 2016, Chen [27] noted 78.04% of the anatomical success rate for AlloCLET, and 1 year later, Cheng [28] noted an almost identical result (78%), following 41 and 80 eyes, respectively, for 12 months. However, Borderie [29] noted very low success rates (29% at 36 months and 0% at 60 months). Indeed, the number of patients followed was only seven. While Campbel [30] found no donor DNA on the ocular surface and no visual improvement, Wang [31] in the same year, 2019, found a 71% anatomical success rate for AlloCLET.

### 4.3. Concept Improvement

Ocular surface surgeries could be made easier, as proved by Subramaniam et al. [32] who cultivated on the same plate, onto AM, LESCs, and conjunctival epithelium, separated by a 360 degrees ring. This coculture was successful in re-establishing both the corneal and conjunctival surfaces in one procedure, in 63% of the eyes.

A clinical trial from Australia [33] investigated alternative substrates for cell cultivation. An already approved biomaterial, a silicon-hydrogel contact lens was used as a carrier, together with a xeno-free culture medium, Eagle medium with 10% autologous serum and antibiotics, providing readily available materials for successful cultivation of epithelial cells. LESCs were harvested from the contralateral eye in unilateral LSCD, while conjunctival biopsies were performed in bilateral conditions. Contact lenses carrying cultivated cells were placed onto deepithelialized cornea. In addition, 25% of the cultures were unsuccessful, requiring a second biopsy. Moreover, 75%, 69%, and 63% of the corneas were stable, at 12, 24, and 30 months, respectively. The source of epithelial cells (limbal or conjunctival) did not statistically influence the survival rates of the graft. Fifty percent of the failures had previous conventional grafts (for LSCD) that had also failed. Conjunctival epithelium seems to be a promising source for corneal reconstruction. The exact mechanism of how conjunctival cells repair the cornea is not fully understood, but there is the possibility of conjunctival own stem cells that transdifferentiate while being exposed to corneal signals.

Baradaran-Rafii [34] added AM extract eye drops to the conventional SLET technique. In short, AM drops were prepared from frozen AM (using nitrogen liquid), which was grounded, liquefied, and sterilized through filtration. The authors also used a smaller piece of donor tissue (2 by 1 mm) than standard SLET (2 by 2 mm). The graft was then sutured at the superior limbus. Corneal conjunctivalization and vascularization regressed 2–3 months after the transplant in all cases. Persistent epithelial defects were observed in all controls. AM extract eye drops could stimulate the cell expansion over the substrate.

### 4.4. Outcome Report

Although there is an impressive amount of clinical data regarding the use of LESC for the treatment of ocular surface, there is an inconsistency regarding the outcome report. While some authors followed the presence of donor DNA on the ocular surface, others investigated the corneal phenotype on impression cytology, and others described the ocular surface or the visual outcome. Shortt [35] correlated the outcomes regarding BCVA with biomarkers of the ocular surface. A clinical improvement in corneal haze, vascularization, and epithelial irregularity correlated significantly with an improvement in visual acuity. Epithelial integrity, although a critical sign of LSCD, did not correlate statistically well with clinical improvement.

Pellegrini [36] noted the success of CLET, using fibrin cultivated LESC explant, in 66.05% of 152 patients, at 12 months (total follow-up averaged 92 months). Full success meant a transparent, avascular, and stable corneal surface had been restored, and was recorded in seven patients. In 12 patients with partial success or failure, a regraft was possible with cultures prepared from frozen original biopsy or from a new biopsy. The long-term follow-up in this multicentric study showed that surgical and cultivation procedures were safe and effective. Debates in the field mostly relate to the definition of biological parameters instrumental for the clinical success of grafts; unambiguous identification of stem cells contained in the cultures; stem cell engraftment and mode of action; and variability of clinical results. The authors also conclude that the most important biological criterion to assess graft quality and the likelihood of a successful treatment is a rather precise evaluation of the number of stem cells detected of holoclones (existing in a small percentage in the explant), expressing high levels of p63 transcription factor, a determinant of the regenerative potential of epithelial stem cells.

For Zakaria [37], the definition of anatomical success is the reversion to a persistent intact epithelium, while the functional success is the significant improvement in pain, photophobia, and BCVA. In 18 patients followed-up for 22 months, on average, the anatomical success was observed in 67%, while the functional success was not achieved in any of the patients. LESC transplantation should not be considered a sight-restorative procedure, the authors concluded.

Pedrotti [38] observed moderate to substantial agreement between IVCM and slit-lamp biomicroscopy or IC analysis in the evaluation of outcomes, performing analysis before and 12 months after CLET, in 13 eyes. There was an 84.5% concordance between clinical outcome (whether the cornea is transparent, avascular, stable or not), and IVCM. IVCM and IC showed 77% concordance. In addition, 76.9% of the cases were classified as total (avascular, transparent, stable epithelium) or partial (recurrence of superficial neovascularization) clinical success, while 46.1% demonstrated central corneal phenotype on IVCM.

Zakaria [39] pointed out the utility of intraoperative anterior surface OCT, to provide the real-time feedback on corneal structure during CLET. Moreover, OCT allows observing the integration of transplanted tissue onto the corneal surface.

Corneal epithelium and clinical success of the stem transplantation could be assessed with IC and IVCM, as also suggested by Prabhasawat in 2019 [40]. Clinical appearance—slit lamp biomicroscopy, IC, and IVCM showed a good correlation, with observed agreement about 80%.

In 2019, Behaegel et al. [41] published long time follow-ups after CLET, 6.9 years on average for 13 patients. Noticing an increase in anatomical failure over time (the success rate dropped from 46.1% on short term to 23.1% on long term), they made the supposition that anatomical and functional results of CLET may decrease over time and question the long-time effect of CLET, in general. A gradual loss of the transplanted cells over time may account for the decrease in clinical outcomes. It seems that there is no evidence that the normal limbal niche features reform after limbal tissue transplantation [38].

### 4.5. Paediatric Population

The first report on paediatric CLET (xeno-free, explant culture technique) came in 2013 from Sejpal [42], who performed the surgeries on 107 children up to 15 years of age, with a mean follow-up of 3.4 years. In addition, 37.4% achieved completely epithelialized, avascular, and stable ocular surfaces. Moreover, 31.8% of the primary failed cases underwent a second CLET, with a 29.4% success rate. Ocular surface stability was achieved in 50 cases (46.7%), at the end of follow-up, after one or two CLET. The mean duration between the injury and CLET was 15.5 months. Furthermore, 54.2% of the eyes had improvement in visual acuity of 0.2 or more logMAR units.

Basu [43] performed SLET procedures on 125 cases, among them 65 children, for an average follow-up of 18 months. One clock-hour wide explants were placed onto AM with fibrin glue scaffold. The outcomes were similar in pediatric (*n* = 65) and adult (*n* = 60) groups, 72% and 80%, respectively showing completely epithelized, stable, and avascular corneal surface. At least two lines of increase in BCVA was observed in 72% of cases. The donor, fellow eye showed no complications or LSCD. The immunohistochemistry showed positive for p63 marker in SLET corneas, as well as CK3 and CK12, while conjunctival markers, MuC5AC and CK19, were absent.

### 4.6. Mesenchymal Stem Cells

In 2018, Calonge [44] conducted a randomized, double-masked pilot trial to prove the efficacy and safety for allogeneic bone marrow-derived mesenchymal stem cell transplantation (MSCT) for LSCD. They compared 17 patients with MSCT versus 11 patients with the CLET procedure. Global success at the end of follow-up (12 months) was found similar for the two groups (around 80%). Central corneal epithelium improved in 71.4% in MSCT, similar with CLET, 66.7%. Allogeneic MSC can be transplanted without the need of host immunosuppression, while allogeneic transplantation of limbal epithelial stem cells requires 1 year of systemic immunosuppression to avoid immune rejection. In this study, however, immunosuppression was administered in both groups. No immune rejection was recorded, and the adverse events were unrelated to cell transplantations.

### 4.7. Stromal Reconstruction

Alio del Barrio [10] investigated alternative sources of stem cells in an attempt to restore the integrity of the stroma. A small number of patients with keratoconus stage IV (*n* = 5), received ADSC (extracted from liposuction fat) in a concentration of 3 × 10^6^ per eye. Injection inside the stroma was assisted by femtosecond laser performing a lamellar dissection 9.5 mm in diameter at about 50% in depth. No complications were recorded, while the visual acuity was improved by two lines in all patients, at 6 months. Corneal transparency was fully recovered at 24 h after the procedure and remained the same through follow-up. In 2019, Alio del Bario [45] evaluated the 1-year safety and efficacy of transplanted ADSC, with (*n* = 5) or without decellularized corneal laminas (*n* = 5), in keratoconus patients. No complications were observed, and corneal transparency recovered within 3 months after the transplant.

The approach was continued by the team, and El Zarif in 2020 [9] investigated ADSC stromal injection versus decellularized human corneal stroma injection (with and without the ADSC supplement). IVCM allowed the observation of the evolution of implanted mesenchymal stem cells. Implantation of ADSC alone allowed for a statistically significant increase in the density of corneal keratocytes in the anterior, mid-, and posterior stroma. However, even the decellularized human corneal stroma, whether impregnated with ADSC or not, favoured an increase in the number of corneal stromal cells in the anterior and posterior stroma of the cornea in a highly statistically significant level. The increase of keratocyte number was higher when ADSC had been added.

### 4.8. COMET and CAOMECS

Ocular surface reconstruction in bilateral LESCD is a challenge. In 2004, Nishida et al. [46] harvested 3 × 3 mm specimens from oral mucosa and fabricated cell sheets on amniotic membrane. COMET or cultivated autologous oral mucosa transplantation was proven successful in all four patients.

Nakamura et al. [47] used the same technique. Fifty three percent of 19 eyes improved in visual acuity at 36 months after the transplant, while seven eyes (37%) were documented with at least one episode of persistent epithelial defect during the follow-up period. Satake et al. [48] also found 53.1% success rate at a mean follow up of 25.5 months. In 2013, Sotozozo [49] observed between 20%–50% success rate in 46 eyes, in the long-term, depending on the ethiology of LSCD.

In 2012, Burillon et al. [50] further developed the concept, without the amniotic membrane, resulting in CAOMECS that use a novel temperature-responsive culture well. The resulting sheet can be transplanted without the substrate, which could cause corneal opacity. They followed 26 transplanted eyes for 12 months, with a success rate of 75%. Characterization of tissue-engineered epithelial cell sheets showed the expression of cytokeratin 3 and p63, a putative marker of stem cells. In 2014, Kocaba et al. [51] observed a success rate of 62.5% (corneal epithelium of a greater quality). They also performed an immuno-histological analysis. One year after CAOMECS, nearly all the basal cells expressed p63 marker, proving the regenerative capability. However, regarding the success rate, Dobrowolski [52] had similar results using COMET (76.4%).

In 2017, Kim et al. [53] introduced COMEC or biomaterial-free cultured oral mucosalepithelial cell sheets. Cultivated sheets are detached from the culture dish using dispase. The clinical trial showed a 75% success rate to reconstruct the cornea and a two-line improvement in BCVA in 62.5%.

### 4.9. Retina

Any structure inside the eye could be someday reconstructed from stem cells progenitors. A target nowadays is the reconstruction of retina. This structure is far more complex than the cornea, and it is in fact a part of the central nervous system. Retinal stem cell therapy targets diseases with a huge impact on the quality of life and highly prevalent in the population. Age related macular degeneration (AMD) represents the main cause of vision loss in developed countries. The increasing life expectancy and lack of efficient treatment, makes AMD, in either of its forms, wet or dry, an immense burden to society. Other diseases targeted with stem cell therapies are mostly Stargardt disease and retinitis pigmentosa.

Different approaches try to replace the retinal pigment epithelial cells (RPE) or even retinal cells. Retinal nerve cells derived from Muller stem cells, human embryonal stem cells (hESCs) or induced pluripotent stem cells (iPSC) are investigated, with no clinical trials finished so far [54].

Masayo Takahashi and Yasuo Kurimoto [55] pioneered the retinal reconstruction using iPSC, and in 2015, the first case of wet AMD received autologous iPSC. Later, they also used allogeneic iPSC. Starting in 2016, there is an ongoing trial, yet to publish results.

### 4.10. Suprachoroidal Stem Delivery

The Cuban approach of Pelaez [56], starting in 1992, involved implanting a pedicle flap of retrobulbar fat with blood vessels, under the sclera, to bring additional blood produced by the transposed vessels to the suprachoroidal space. Increased blood flow in patients with retinitis pigmentosa would stabilize the disease. Electrostimulation and ozone therapy would add to the benefit. The procedure brought criticism from the international community [57], independent reports observing no improvement in visual function or ERG.

In the Limoli variant [58], the distance between the adipose flap (harvested from the area of the inferior oblique muscle) and choroid was reduced, by the mean of a deeper sclerectomy (infero-temporal, 8 mm from the limbus), the area of the graft was expanded (5 by 5 mm), ADSCs from stromal vascular fractions were added, together with platelet rich plasma (through centrifugation of the blood material, separation of the component, and its platelet degranulation). The changes would increase the survival of the graft and trigger the proliferation of the ADSCs, promote the growth of the choroid perfusion, and add a better modulation of the action of the growth factors from the adipose tissue.

Limoli et al. applied Limoli Retinal Restoration Technique (LRRT) in 2014 [58] and treated 12 eyes (12 patients) with an average age of 71.25 years (62–80). Inclusion criteria were white race, well nourished, AMD confirmed (OCT, autofluorescence, fluorescein angiography), good extrafoveal area, measurable VA, and normal IOP. Exclusion criteria were exudative AMD, myopia > −6D, ocular disorders (cataract, glaucoma, neovascular disorders, etc.) and insufficient compliance. The baseline evaluation included BCVA (ETDRS logMar) and ERG. The outcome showed an increase in scotopic rod-ERG (47.44%, *p* < 0.05), scotopic maximal rod-cone ERG (15.56%, statistical non-significant, *p* < 0.1), photopic cone-ERG (13%, statistical non-significant). BCVA remained unchanged (0.78 logMar). An increase in electrical response of the extrafoveal area was observed, in the short-term (30 days), with no change in foveal activity, possibly due to the already reduced number of cones in this area. No mERG was possible in this study. Cell-mediated therapy could improve the electrical cell response through growth factors. No adverse reaction was reported.

In 2018, Limoli et al. [59] performed 11 transplantations on dry AMD, using the LRRT technique, placing ADSCs harvested from abdominal fat, platelets derived from PRP, and adipose pedicle, into the suprachoroidal space. This was a case-control study, randomizing 25 eyes into two groups: 11 eyes treated and 14 eyes control. In the treated group, BCVA improved from 0.581 to 0.376 in 6 months (*p* < 0.01), compared to the untreated group, where BCVA worsened non-significantly, from 0.573 to 0.601 logMAR. No complications were observed (no sub-retinal neovascularization, macular edema or retinal detachment).

### 4.11. Intravitreal Stem Delivery

In 2011, Siqueira [60] performed intravitreal injection with BMSC. Five patients (three with RP and two with cone-rode dystrophy), with BCVA < 20/200, received 10 × 10^6^ autologous bone marrow–derived mononuclear cells (0.1 mL) into one eye. Four patients showed a one-line improvement of BCVA from 1 week after injection to the end of the study, at 10 months. No adverse reaction was observed.

In 2015, the same research group [61] investigated the quality of life of the patients with retinitis pigmentosa receiving intravitreal BMSC. In addition, 0.1 mL of cell suspension was used for the intravitreal injection, containing 0.92 × 10^4^ to 2.91 × 10^4^ bone marrow–derived hematopoietic stem cells (CD34+). Twenty patients received the injection in the worse eye. The survey included a questionnaire (National Eye Institute Visual Function Questionnaire), which showed a temporal improvement of the quality of life at 3 months, whereas at 12 months after the treatment the benefit was lost. The improvement at 3 months could have been induced by the patient expectations.

In a study from 2014, Park et al. [62] injected bone marrow stem cells (CD34+) into the vitreous, in various diseases leading to irreversible loss of vision (AMD two cases, retinitis pigmentosa one case, retinal vascular occlusion one case, and Stargardt two cases). The average dose was 3.4 million CD34+ cells per eye. The procedure appeared safe, with no proliferation or inflammation. BCVA improved 10 letters in four out of six eyes, at 6 months. In one Stargardt patient, the high-resolution OCT observed fine hyperreflective deposits into the retina, 1 month after the injection, in which the authors hypothesis to be CD34+ cells.

### 4.12. Subretinal Stem Delivery

In 2015, Schwartz et al. [63] described two-phase, one prospective clinical study, investigating the role of hESCs-derived RPE for AMD (nine patients) and Stargardt (nine patients). Both were registered with ClinicalTrials.gov. Three doses were transplanted: 50,000, 100,000, and 150,000 cells (each for three patients with AMD and Stargardt). Immunosuppression was needed, with Tacrolimus and Mycophenolate mofetil 1 week prior to surgery and 12 weeks after. BCVA improved a median of 14 letters at 12 months in 10 eyes (with similar results for the two diseases), remained mostly stable in seven eyes, but decreased in one eye. In the fellow untreated eye, there was no similar change in BCVA. The change in BCVA was not significantly influenced by the number of cells transplanted. There was no evidence of adverse proliferation, rejection or serious ocular or systemic safety issues related to the transplanted tissue. Subretinal pigmentation was observed in 13 cases, consistent to the transplanted RPE. One eye developed endophthalmitis (*Staphylococcus epidermidis*), one eye had a mild epiretinal membrane, and four eyes required cataract surgery. Follow-up was continued for at least 12 months, with a median of 22 months.

In 2016, Oner et al. [64] focused on the delivery of ADSCs into the subretinal space in advanced stage retinitis pigmentosa, through 23G pars plana vitrectomy (PPV). In addition, 47 × 10^6^ ± 0.11/150μL of ADSC suspension was injected with a 41-gauge needle, far from fovea. From 11 eyes, only one showed improvement in BCVA (from 20/2000 to 20/200), visual field, and ERG. The study faced numerous complications (six patients)—neovascular membrane at the site of implantation (one case) and epiretinal membrane around the transplantation and in the periphery (five cases), receiving a second vitrectomy. However, only one donor was used for the harvesting of ADSC, with the purpose of eliminating donor-based differences, but hence increasing the immunologic imbalance at the site of injection. The authors advised caution when performing subretinal stem cells transplantation of ADSC. Optimization of cell delivery while maintaining the retinal pigment epithelium integrity could yield better results, maintaining the immune privilege of the eye.

Oner et al. changed the retinal approach, from internal to external (suprachoroidal), and, in 2018, [65] investigated ADSCs use for the treatment of four eyes with atrophic AMD and four eyes with Stargardt, followed up for 6 months. LRRT was performed this time. The worse eye was treated. All the patients experienced improvement in the treated eye, regarding BCVA and visual field, without similar results in the untreated eye. The mERG showed improvement in P1 waves. Fluorescein angiography remained unchanged. On the OCT, macular thickness increased from 108 to 129 microns, while the choroid showed increased thickness (259 to 298 microns). Suprachoroidal implantation of stem cells seemed safe, with no complications after 6 months.

In 2017, Liu et al. [66] cultivated retinal precursor cells (RPC) from ocular tissue collected in tissue banks, originating from aborted foetuses 12–16 weeks old. RPC were isolated and cultivated from the neural retina. Through pars plana vitrectomy, a small quantity of 100 μL containing roughly 1 × 10^6^ cells were injected through a 39G canula, into the subretinal space. BCVA improved between 2 and 6 months after the surgery (*p* < 0.05), but slowly declined afterwards, with no difference from baseline observed at 24 months. The ERG and visual field were unreliable in this study, due to the low vision of the patients. Allogeneic transplantation of human fetal-derived RPC was safe and did not produce any rejection, inflammation or neovascularization.

In 2018, Mehat [67] investigated the use of subretinal hESC-derived RPE cells (from hESC line MA09) in 12 patients with Stargardt dystrophy. Systemic immunosuppression was needed for 13 weeks. Four doses were administered in each group of three patients: 50,000, 100,000, 150,000, and 200,000 cells. There was no improvement in BCVA and Microperimetry at 12 months. Moreover, the authors found no change in quality of life (National Eye Institute Visual Function Questionnaire). Subretinal pigmentation was observed in all cases, which may be a sign of survival of transplanted RPE cells. There was no sign of proliferation, inflammation or immune rejection. Five patients had reactions to immunosuppression.

### 4.13. Other Concepts

The stem cell ophthalmology treatment study (SCOTS) is a NIH registered clinical study (NCT03011541) [68]. It is an interventional clinical trial that started back in 2012, with 300 cases enrolled, follow-up for at least 12 months, with the purpose of evaluating the use of autologous bone marrow derived stem cells (BMSC) for the treatment of retinal and optic nerve damage or disease. The comparator is the natural history of the disease. BMSC were injected retrobulbar (RB), subtenon (ST), intravenous (IV), intravitreal (IVIT), and intraocular (intravitreal or subretinal) with vitrectomy, in a progressive approach, depending on the severity of the disease. The completion date was July 2020, with only preliminary results published so far—Table 3 [69,70,71,72,73,74,75]. The primary outcome was the change of BCVA measured with Snellen and EDTRS charts. The secondary outcome was the change in visual field (automated perimetry). Patients over 18 years of age, with visual acuity less than 20/40, and/or abnormal visual field with objective, documented damage to the retina or the optic nerve, progressive or unlikely to improve, were included. Since BMSC are autologous cells, no immunosuppression was necessary. Clinical improvement ranged from 63% to 100% in various diseases: AMD, retinitis pigmentosa, Usher syndrome, Leber neuropathy, dominant atrophy, and nonarteritic ischemic neuropathy. For AMD, BCVA increased 26.7% in logMAR units. Possible mechanisms by which improvement occurred may include transdifferentiation of BMSC into Neuronal Nuclei positive cells, BMSC paracrine secretions or neurotrophic factors and hormones, transfer of mitochondria, release of messenger RNA or other compounds via exosomes or microvesicles.

In 2018, Kashani et al. [76] conducted a prospective phase I clinical trial, assessing the safety and efficacy of a subretinal composite implant (California Project to Cure Blindness–Retinal Pigment Epithelium 1)—a polarized monolayer of Human embryonic stem cell–derived RPE on a nonbiodegradable, synthetic parylene substrate (that mimics the Bruch membrane), 3.25 by 6.25 mm. The implant was delivered into a subretinal pocket created with a 41G infusion needle followed by the hydrodisection of the retina overlying the geographic atrophy, during a 23G PPV, and using a custom-made forcep. The implant had to be inserted through an enlarged 20G sclerotomy. The results support the safety, anatomic integration, and functional activity of this implant as a potential treatment for severe vision loss from Nonexudative AMD. Follow-up varied from 4 to 12 months. One eye could not be implanted, while the others showed good results in terms of BCVA (one substantial improvement of 17 letters LogMar, three eyes without progression of loss). The only event reported was the retinal haemorrhage (mild to moderate in most cases, one subretinal necessitating one injection of Bevacizumab).

### 4.14. Perspectives

The medical literature is abundant in research regarding corneal stem cells. The clinical implementation is also far ahead compared to stem cells therapy for the retinal diseases. However, only in 2019, we have the first randomized clinical trial concerning AlloCLET in severe bilateral limbal stem cell deficiency to demonstrate the feasibility and safety of this approach [30]. The authors noticed an improvement in ocular surface score (OSS, with corneal parameters regarding the epithelium, hyperemia, neovascularization, opacification, and conjunctivalization, ranging from 0 = normal to 3 = severe damage). While OSS is improved in both the stem cells treated group and AM treated group, the effect is sustained at 18 months in the stem cells group, only. The effect seems unrelated to the long-term survival of donor cells, as there was no donor DNA found in the graft at 6 months. The early effect of donor stem cells could benefit the health of the ocular surface, in an unknown mechanism, possibly related to the stimulation of the small number or dormant stem cells in the host’s niche.

The complexity of limbal stem niche could someday be recreated. Levis and Daniels [77] created an in vitro model, a bioengineered limbal crypt, with three-dimensional features to maintain an immature population of LESC that express typical markers of stem cells.

The future could see artificial corneas. In fact, a clinical trial (phase I) started in Spain in 2017 [78], investigating the safety and feasibility of a nanostructured fibrin-agarose corneal substitute combining allogeneic cells, that mimics the anterior human native cornea. Artificial corneal substitute, that can be cultivated with cells, could be an ideal therapeutic approach for patients with severe damaged corneas and LSCD.

Apart from cornea and retina, other structures of the eye could someday be replicated, starting with stem cells. The iPSC can be induced to resemble native trabecular meshwork cells [79]. For retinal reconstruction, the conjunctiva could be a better source of somatic adult cells for deriving iPSCs [80].

In 2019, Nishida reported the first transplant of epithelial sheet derived from iPSCs. With four transplants planned in total, the clinical study is set to be finished in 2021 [81].

Delivery of stem cells under the retina is a difficult process. Early trials used cell suspensions injected under the retina and showed low RPE attachment and survival rates. Transplanted sheets of stem cell derived RPE are carried on a matrix that will suffer degradation that may injure the retinal environment. Parylene, polyesters, and poly glycolic acid-lactic acid (PLGA) have been used as a support. The rigidity of the materials and slow degradation carry a risk for the choroid and RPE, causing fibrosis and inflammation. Fibrin hydrogels have been proposed lately, suffering a faster degradation and less adverse effect to the cells [82].

Retinal stem cell research entered a clinical phase. Various teams have registered clinical trials on clinicaltrial.gov, regarding the use of stem cells in retinitis pigmentosa, macular degenerations and dystrophies, optic neuropathy, primary open angle glaucoma, and central retinal vein occlusion. Many of these studies are still recruiting, and most of them have no results yet.

## 5. Conclusions

This regenerative medicine holds great promise regarding the reconstruction of various structures of the eye. So far, corneal epithelial reconstruction seems well validated clinically. Enough clinical data are collected to allow some form of standardization of the stem cell transplant procedures. In comparison, far less patients are investigated in retinal stem reconstructions, so far, with lower anatomical and clinical success. Many clinical studies are underway and explore intravitreal cell suspension, subretinal cell suspension, subretinal stem on matrix, and suprachoroidal techniques. RPE replacement, photoreceptor rescue or even photoreceptor replacement are based on a technology to be yet refined.

## Figures and Tables

**Table 1:** Summary of studies involving corneal reconstruction. Study concept and outcome (anatomical and functional).

Author, Year	Study Type, Follow-Up (Mean)	No. of Patients (Eyes)	Pathology Targeted	Concept	Surgery—Substrate, Technique	Anatomical Outcome	Visual Outcome
Pauklin 2010	Consecutive, case series, 28.5 months	38 (44)	Burns, Pterygium, Aniridia, Perforating eye injury, Mitomycin C, Epidermolysis bullosa	CLET	LESC on AM, autologous or allogeneic cells	68.2% success - outcome better in autologous versus allogeneic transplantation (76.7% vs. 50%, *p* < 0.05) - outcome better in partial versus total LSCD (83.3% vs. 63.3%)	72.7% of eyes, improvement (average 8.7 lines)
Nakamura 2011	Prospective, 36 months	17 (19)	Burns, Pemphigoid	COMET	Oral mucosa cultivated on AM	100% success rate	BCVA improvement (hand movement to 20/40) 53%
Marchini 2011	Case series, 12 months	16 (16)	Chemical burns	CLET	LESC cultivated on AM	62.6% success rate	NA
Sangwan 2011	Retrospective, 36 months	200 (200)	Burns (unilateral)	CLET	LESC cultivated on AM (explant), xeno-free	71% corneal phenotype No complications for donor site	60.5% of eyes, two lines improvement
Satake 2011	Retrospective, 25.5 months	36 (40)	Stevens Johnson, Burns, Pemphigoid	COMET	Oral mucosa cultivated on AM	53.1% success rate Corneal melting or perforation in eight eyes	NA (possible improvement)
Basu 2012	Retrospective, 21 months	50 (50)	Failed CLET, after Ocular Burns (unilateral)	CLET	LESC cultivated on AM (explant), xeno-free	66% corneal phenotype No complication for donor site	76% of eyes, improvement (two lines)
Basu 2012	Retrospective, 56 months	21 (28)	Bilateral, total LSCD	AlloCLET	Allogeneic LESC cultivated on AM, xeno-free	71.4% success rate	67.8% of eyes, improvement at least to 20/60
Burillon 2012	Prospective, 12 months	25 (26)	Burns, Aniridia	CAOMECS	Cultured autologous oral mucosa, 3 × 3 mm biopsy	75% success rate one perforation one rejection	NA
Sangwan 2012	Case series, 9 months	6 (6)	Burns	SLET	2 × 2 mm explant, divided and placed onto AM	100% success rate	66% of eyes, improvement
Pelegrini 2013	Prospective, multicentric, 96 months	152 (152)	Burns, keratitis, contact lens abuse—unilateral LSCD	CLET	LESC-fibrin-cultured (explant)	66.05% total success at 12 months	NA
Qi 2013	Retrospective, case series, 12 months	41 (42)	Burns	AlloCLET	LESC cultivated on AM	76.2%—stable cornea 23.8% rejection	NA
Sotozono 2013	Prospective, 85.6 months	40 (46)	Burns, Stevens Johnson, Pemphigoid	COMET	Oral mucosa on AM	20%–50% 40% persistent epithelial defect	Improved BCVA ½ lines: 50%—SJS, 20%—burns, 49% pemphigoid
Sejpal 2013	Retrospective, interventional, case series, 41.2 months	107 (107)	Chemical or thermal burns	CLET	LESC cultivated on AM	46.7% success rate	54.2% of eyes, 0.2 or more logMAR, improvement
Subramaniam 2013	Clinical study, 33 months	39 (40)	N/A—LSCD and symblepharon	CLET + conjunctival tissue coculture	LESC and conjunctival cells co-cultivated on AM	60% survival rate at 12 months 45% survival rate at 48 months	NA
Kocaba 2014	Prospective, clinical trial, 28 months	22 (23)	Burns, aniridia	CAOMECS	Cultured autologous oral mucosa, 3 × 3 mm biopsy	62.5% success rate (epithelium quality)	BCVA increase 74%
Shortt 2014	Case series, 36 months	17 (18)	aniridia, Stevens Johnson (bilateral)	AlloCLET	LESC cultivation on AM (explant) Immunosuppression		79% of eyes at 6 months, 57% at 24–36 months, improvement
Zakaria 2014	Phase I, clinical trial, 22 months	18 (18)	Burns, aniridia	CLET Autologous *n* = 15 Allogeneic *n* = 3	LESC cultivated on AM, xeno-free	67% success rate	Stable
Bobba 2015	Prospective, clinical trial, 30 months	16 (16)	Chemical burns, aniridia, contact lens abuse, trachoma, iatrogenic	CLET (*n* = 7) Conjunctival epithelium (*n* = 9)	Cultivation on silicon-hydrogel contact lens, Eagle-autologous serum (xeno-free medium)	63% success rate	NA
Dobrowolski 2015	Prospective, 18 months	13 (17)	Aniridia	COMET	Oral mucosa on AM	76.4% success rate	BCVA increase (0.05 to 0.1) 88.2%
Pedrotti 2015	Prospective, interventional, noncomparative, masked, case series, 12 months	13 (13)	Chemical burns, herpes simplex infection	CLET	LESC cultivated on 3T3 and fibrin glue	IVCM—46.2% corneal epithelium	NA
Ramirez 2015	Prospective, noncomparative, case series, 36 months	19 (20)	Burns, Stevens Johnson, pemphigoid, aniridia, keratitis, postsurgical	CLET Autologous *n* = 11 Allogeneic *n* = 9	LESC cultivated on AM	80% at 24 months 75% at 36 months 90.9% autografts 66.7% for allografts	NA
Zakaria 2015	Prospective, observational, case series, 10.6 months	8 (8)	burns, aniridia, Kniest syndrome, microphtalmia, iatrogenic	CLET	LESC cultivated on AM	OCT—100% presence of an epithelial layer	NA
Basu 2016	Prospective, interventional, case series, 18 months	125 (125)	burns	SLET Moreover, children (*n* = 60)	Explant on AM, fibrin glue	76% success rate	75.2% of eyes, two lines improvement
Chen 2016	12 months	41 (41)	burns, trauma, SJS	AlloCLET	Allogeneic LESC cultivated on AM	78.04% success rate	NA
Kaliki 2016	Non-randomized, case series, 12 months	7 (7)	Ocular surface neoplasy	SLET	Wide excisional biopsy, SLET	No LSCD	NA
Parihar 2016	Prospective, interventional study, 12 months	40 (50)	Chemical/thermal burns, Stevens Johnson, Pemphigoid, Chronic ocular allergy	AlloCLET, *n* = 20(25) KLAL, *n* = 20(25)	Allogeneic LESC cultivated on AM, Allogeneic Keratolimbal allograft	Ocular surface restoration similar in both groups	improvement, similar in both CLET and KLAL
Vazirani 2016	Retrospective, multicenter, case series, 12 months	68 (68)	Burns, keratitis, surgery	SLET	1–2 clock hours limbus biopsy, 10–15 pieces distributed onto AM, fixed with fibrin glue	83.8% success rate	64.7% of eyes, two lines improvement
Alio del Barrio 2017	Phase I, 6 months	5 (5)	Keratoconus	Stromal injection of ADSC	Auto ADSC resulted from liposuction	IVCM—survival of ADSC	100% of eyes, two lines improvement
Baradaran-Rafii 2017	Prospective, interventional, controlled, case series 18 months	20 (20)	Chemical burns	SLET + AM extract eye drops (14 cases) SLET only (six controls)	Contralateral graft, sutured onto AM, contact lens, tarsoraphy	Ocular surface improvement in all treated cases Persistent epithelial defect in all controls No complications for donor site	20/400 to 20/50, improvement
Cheng 2017	12 months	80 (80)	Burns	AlloCLET	Allogeneic LESC cultivated on AM	78% success rate	NA
Iyeer 2017	Retrospective, 10.28 months	17 (18)	Chemical burn, grade 4 (Dua)	AlloSLET	AM Cells fixed with fibrin glue and contact lens	13/18 reepithelization seven gradual failures	NA
Kim 2017	Prospective, 15 months	8 (8)	Burns, Stevens Johnson	COMEC	Oral mucosa, biomaterial-free	75% success rate	BCVA improvement (two lines) 62.5%
Prabhasawat 2018	Prospective, multicentric, 12 months	9 (10)	LSCD	SLET	LESC—autoSLET (*n* = 5) and living relatives’ source alloSLET (*n* = 5)	70% success rate three failures (one autoSLET, two alloSLET)	NA
Behaegel 2019	Case series, 79 months	13 (13)	Aniridia, burns, microphthalmia	CLET (*n* = 9) AlloCLET (*n* = 4)	LESC cultivated on AM (explant), xeno-free	23.1% success rate at 79 months (46.1% at 25 months)	NA
Borderie 2019	Case series, 60 months	7 (7)	Burns, aniridia, surgeries	AlloCLET	Allogeneic LESC cultivated on AM	29% success rate at 36 months 0% success rate at 60 months	NA
Calonge 2019	Randomized, double-masked, clinical trial, 12 months	28 (28)	Burns, aniridia, Stevens Johnson, pemphigoid, keratitis	MSCT (*n* = 17); AlloCLET (*n* = 11)	Allogeneic MSC cultivated on AM Allogeneic LESC cultivated on AM	85.7% success rate for MSCT = 77.8% success rate for CLET, Central corneal phenotype 71.4% MSCT = 66.7% CLET	NA
Campbell 2019	Randomized, control trial, controlled multicentric study, 18 months	13 (13)	Bilateral LSCD: Aniridia, chemical burn, pemphigoid, connective tissue disorder	AlloCLET	Allogeneic LESC cultivated on AM (treated, *n* = 7) versus AM alone (control, *n* = 6) Systemic immunosuppression	No donor DNA at 6, 12, and 18 months. OSS improvement	improvement is statistically insignificant
Wang 2019	23.3 months	41 (41)	Burns	AlloCLET	Allogeneic LESC cultivated on AM	71.4% success rate	NA
El Zarif 2020	Interventional, prospective, randomized, 12 months	14 (14)	Keratoconus	Stromal injection of ADSC and corneal decellularized laminas	AutoADSC (*n* = 5) Decellularized human corneal stroma (*n* = 5) AutoADSC + decellularized human corneal stroma (*n* = 4)	Increased density of cells No fibrous tissue	NA

ADSC—adipose-derived MSCs; AlloCLET—Allogeneic CLET; AM—Amniotic membrane; BCVA—best corrected visual acuity; CAOMECS—cultivated autologous oral mucosa epithelial cell-sheet; CLET—Cultivated limbal epithelial stem cells COMET—Cultivated oral mucosal epithelial sheet transplantation; IVCM—in vivo confocal microscopy LESC—Limbal epithelial stem cells; KLAL—Keratolimbal graft; LSCD—limbal stem cells deficiency; MSCT—Mesenchimal stem cells transplantation; NA—not available; SLET—Simple limbal epithelial transplant; SJS—Stevens-Johnson Syndrome.

**Table 2:** Summary of studies involving retinal reconstruction. Study concept and outcome (anatomical and functional).

Author, Year	Study Type, Follow-Up	No. of Patients (Eyes)	Pathology Targeted	Location, Surgery	Type of Cells	Clinical/Anatomical Outcome	Visual Acuity Outcome
Siqueira 2011	Phase I clinical trial, 10 months	5 (5)	Retinitis pigmentosa cone-rod dystrophy	Intravitreal	Bone marrow stem cells	NA	80%—four eyes—one line improvement in BCVA
Limoli 2014	Prospective, observational, 30 days	12 (12)	Atrophic macular degeneration	Suprachoroidal—deep sclerotomy (LRRT)	Adipose tissue, ADSC, platelet rich plasma	ERG increase	NA
Park Susanna 2014	Phase I clinical trial, 6 months	6 (6)	Retinal vascular occlusion Stargardt AMD Retinitis Pigmentosa	Intravitreal, 30G needle	CD34 + bone marrow stem cells	NA	66%—four eyes—BCVA improvement with 10 letters
Schwartz 2015	Phase I, prospective, registered clinical trial, 12 months	18 (18)	AMD Stargardt	Subretinal, 23G PPV	hESC-derived RPE	18 eyes—successful implantation	55%—10 eyes—BCVA improvement at least 15 letters 38%—seven eyes—BCVA stable one eye—BCVA decrease
Siqueira 2015	Clinical study, 12 months	20 (20)	Retinitis pigmentosa	Intravitreal	Bone marrow stem cells	Quality of life improvement at 3 months, no change at 12 months	NA
Oner 2016	Phase I clinical study, 6 months	11 (11)	Retinitis pigmentosa	Subretinal, PPV 23G	ADSC	six complications: One CNV, five epiretinal membrane one improvement (visual field)	9%—one patient—BCVA improvement (from 20/2000 to 20/200)
Liu Y. 2017	Phase I clinical trial, 24 months	8 (8)	Retinitis pigmentosa	Subretinal, PPV	Fetal-derived Retinal progenitor cells	Temporary improvement between 2–6 months, no change in BCVA at 24 months	Stable
Kashani A 2018	Phase I, prospective, interventional, 4–12 months	5 (5)	Advanced nonexudative AMD	Subretinal, 23G PPV	composite subretinal implant	four eyes succeeded the implantation: Three eyes—no progression or vision loss one eye—17 letters improvement	Stable
Limoli 2018	Randomized, case-control, 6 months	11 (11) vs. 14 (14) control	AMD	Suprachoroidal, LRRT	ADSC, adipose tissue, platelet rich plasma	Microperimetry increase (from 11.44 to 12.59 dB)	VA improvement by 32%
Mehat 2018	Phase I clinical trial, 12 months	12 (12)	Stargardt	Subretinal, PPV	hESC-derived RPE	No improvement in BCVA or Microperimetry No change in quality of life No ocular complications	Stable
Oner 2018	Phase I, Prospective, clinical case series, 6 months	8 (8)	Atrophic AMD (four cases) Stargardt (four cases)	Suprachoroidal—deep sclerotomy (LRRT)	ADSC, adipose tissue, platelet rich plasma	Visual field increase, mERG increase, choroid thickness increase (259 to 298)	1.7 to 1.2 LogMar increase

ADSC—adipose-derived MSCs; AM—Amniotic membrane; AMD—age related macular degenescence; BCVA—best corrected visual acuity; CNV—choroidal neovascularisation ERG—electroretinogram; hESC—human embryonic stem cells; LRRT—Limoli Retinal Restoration Technique; MSCT—Mesenchimal stem cells transplantation; NA—not available. PPV—pars plana vitrectomy; RPE—retinal pigment epithelium.

**Table 3:** Stem cell ophthalmology treatment study (SCOTS). Design and outcome (Weiss et al.).

Disease	No. of Patients (Eyes)	Follow-Up	Stem Delivery (Design)	Stem Delivery (De facto)	Clinical/Anatomical Outcome	Visual Acuity Outcome
Dominant optic atrophy	6 (12)	24 months	Arm 1—RB and ST, followed by IV Arm 2—RB, ST, and IVIT, followed by IV Arm 3—for the eye with better BCVA—Arm 1 or 2; for the worse eye—core pars plana vitrectomy, subretinal and intra-optic nerve stem cell concentrate, followed by IV	Arm 1—zero patients Arm 2—five patients Arm 3 + 2—one patient	83.3% of eyes—improvement (five out of six patients; one patient unchanged) Less effect for Arm 3	BCVA increase, 29.5% LogMar
Usher syndrome	5 (10)	12 months	Arm 1—in mild vision loss Arm 2—VA > 20/200 Arm 3—for the eye with VA < 20/200 Arm 1/2 for the better eye	Arm 1—four eyes Arm 2—six eyes Arm 3—zero eyes	80% of eyes—improvement	BCVA increase, 28.3% LogMar
AMD	16 (32)	12 months	Arm 1—if IVIT not possible (silicone oil) Arm 2—VA 20/40-20/200 and/or visual field loss Arm 3—for the eye with VA < 20/200, Arm 1/2 for the better eye	eight patients—Arm 1 in one eye, Arm 2 in the other five patients—Arm 2 in both eyes one patient—Arm 3 in one eye, Arm 1 in the other one patient—Arm 3 + 2	63% of eyes—improvement 34% of eyes—stability at 12 months No statistical difference between Arm 1 and Arm 2 one retinal detachment after 4 months of Arm 2	BCVA increase, 27.6% LogMar
Leber optic neuropathy	5 (9)	12 months	Arm 1, 2, 3—identical to AMD	four patients—Arm 3 + 2 one patient—Arm 2 monocular	100% improvement No complications	Various results (best—from hand movement to 20/200)
Non arteritic ischemic optic neuropathy	5 (9)	12 months	Arm 1, 2, 3—identical	seven patients—Arm 3 + 2 one patient—Arm 2 monocular two patients—Arm 2	73.6% of eyes—improvement (15.9% remained stable) Duration of vision loss did not affect the outcome	BCVA increase, 22.74%
Retinitis pigmentosa	17 (34)	6 months	Arm 1, 2, 3—identical	10 patients—Arm 1 (one of them, monocular) six patients—Arm 2 one patient—Arm 1/Arm 2	64.7% of eyes—improvement (the rest, no change) No complications reported Duration of disease did not affect the outcome	BCVA increase, 40.9%

AMD—age related macular degenerescence; BCVA—best corrected visual acuity; IV—intravenous; IVIT—intravitreal. PPV—pars plana vitrectomy; RB—retrobulbar; ST—subtenon.

## Data Availability

The data presented in this study are openly available international databases: https://pubmed.ncbi.nlm.nih.gov/ (accessed on 20 June 2021), https://clinicaltrials.gov/ (accessed on 20 June 2021).
